# Ontogeny of Numerical Abilities in Fish

**DOI:** 10.1371/journal.pone.0015516

**Published:** 2010-11-24

**Authors:** Angelo Bisazza, Laura Piffer, Giovanna Serena, Christian Agrillo

**Affiliations:** Department of General Psychology, University of Padova, Padova, Italy; Yale University, United States of America

## Abstract

**Background:**

It has been hypothesised that human adults, infants, and non-human primates share two non-verbal systems for enumerating objects, one for representing precisely small quantities (up to 3–4 items) and one for representing approximately larger quantities. Recent studies exploiting fish's spontaneous tendency to join the larger group showed that their ability in numerical discrimination closely resembles that of primates but little is known as to whether these capacities are innate or acquired.

**Methodology/Principal Findings:**

We used the spontaneous tendency to join the larger shoal to study the limits of the quantity discrimination of newborn and juvenile guppies. One-day old fish chose the larger shoal when the choice was between numbers in the small quantity range, 2 vs. 3 fish, but not when they had to choose between large numbers, 4 vs. 8 or 4 vs. 12, although the numerical ratio was larger in the latter case. To investigate the relative role of maturation and experience in large number discrimination, fish were raised in pairs (with no numerical experience) or in large social groups and tested at three ages. Forty-day old guppies from both treatments were able to discriminate 4 vs. 8 fish while at 20 days this was only observed in fish grown in groups. Control experiments showed that these capacities were maintained after guppies were prevented from using non numerical perceptual variables that co-vary with numerosity.

**Conclusions/Significance:**

Overall, our results suggest the ability of guppies to discriminate small numbers is innate and is displayed immediately at birth while discrimination of large numbers emerges later as a result of both maturation and social experience. This developmental dissociation suggests that fish like primates might have separate systems for small and large number representation.

## Introduction

Over the last few decades, basic numerical abilities have been demonstrated for human infants [1], [2], non-human primates [3], [4] and several other vertebrates (mammals: [5], [6], [7]; birds: [8], [9]; amphibians: [10]; fish: [11], [12], [13]) and invertebrates [14], [15].

The evidence collected in comparative and developmental research suggests that adults prevented from verbal counting, infants and non-human primates possess similar numerical capacities [16], [17], [18], [19]. In particular they suggest the existence in human and non-human primates of two distinct non-verbal quantificational systems, one, the small number system, precise but subject to a set size limit of 3 or 4 and one, the large number system, approximate and subject to a ratio limit, i.e. with better accuracy for larger ratio differences (reviewed in [17], [20]). The former has been proposed to depend on a system for representing and tracking small numbers of individual objects [16], [21], [22]. Since it operates by keeping track of individual elements, it is precise but allows for the parallel representation of up to 3–4 elements [23]. For instance, it has been shown that 12-month-old infants are able to select the larger quantity of crackers when the paired numbers are 1 vs. 2 and 2 vs. 3, but fail with 3 vs. 4 and 3 vs. 6 [16]. Similarly, rhesus monkeys, confronted with two quantities of apple slices, successfully choose the greater number with comparison of 1 vs. 2, 2 vs. 3 and 3 vs. 4, but fail with 4 vs. 5 and 4 vs. 6 [19]. In chimpanzee, error rate and reaction time are constant in the range 1–4 while they tend to increase monotonically for larger numbers [24], [25]. The second mechanism is an analog magnitude system for approximate numerical estimation that obeys Weber's Law, which maintains that, as numerical magnitude increases, a larger disparity is needed to obtain the same level of discrimination. Xu and Spelke (2000) demonstrated [2] that 6-month-old infants tested by using the habituation-dishabituation paradigm are able to distinguish between 8 and 16 dots (1∶2 numerical ratio), while they are unable to discriminate closer ratios such as 2∶3 (8 vs. 12 dots). Flombaum and colleagues (2005) found that rhesus monkeys successfully discriminate between 4 and 8 lemons (1∶2 numerical ratio) but not between 4 and 6 (2∶3), indicating a similar limit for monkeys and 6-month-old infants [26].

Not all empirical studies support the existence of a separate cognitive mechanism for representing small sets of objects. vanMarle and Wynn [27] for instance found that infants' discrimination of auditory events was ratio-dependent even for small values, suggesting that infants can use analog magnitudes for both small and large quantities in the auditory domain. Another study reported that rhesus monkeys and adult humans showed a similar performance in a task requiring them to order pairs of numerosities and that accuracy and reaction time were similarly affected by numerical ratio in the large and small number range [28]. To explain this inconsistency, it has been argued that small quantities may be represented by both analog magnitudes and object-files, and the context in which the representation is elicited determines which of the two systems is employed [27], [29].

Despite some indirect evidence suggest distinct systems for large and small numbers might exist in the eastern mosquitofish, feral dog and New Zealand robin [8], [30], [31], no study has yet investigated in vertebrates other than primates whether a single analog magnitude system accounts for discrimination over the full range of numerical values or there is a distinct, precise, system based on object-file for processing of small numbers.

The study of developmental trajectories can be a powerful tool to investigate the functional architecture underlying cognitive processes. If a single system underlies numerical discrimination one would expect the same developmental trajectories for the discrimination of numbers in the small and large ranges. Conversely, a developmental dissociation, either in terms of onset timing or age-related change in performance, would indicate that different systems are probably at work. Presently, longitudinal data are available only for infants and are limited to the discrimination of large numerosities (reviewed in [29]). On the whole, they indicate that this capacity is normally present in 6-month-olds and increases in precision during development. Six-month-old infants can discriminate numerosities with a 1∶2 ratio (such as 8 vs. 16) but not a 2∶3 ratio, whereas 10-month old infants are able to discriminate numerosities with a 2∶3 but not a 4∶5 ratio. The resolution of this system continues to increase throughout childhood, with 6-year-olds being able to discriminate a 5∶6 ratio and adults a ratio of 9∶10 [1], [32], [33], [34]. A recent study investigating numerical cross-modal matching suggests that even two-day-old infants may be able to discriminate quantities but only with a 1∶3 ratio [35]. As regards the small number range, experiments conducted with two different paradigms indicate that 6-month-olds can discriminate 2 vs. 3 items, but no information is available on other numerical contrasts [36]. At 12 months, infants discriminate 2 vs. 3 but not 3 vs. 4 or 2 vs. 4 items [16]. Different paradigms were used to study 6- and 12-month-olds and so results cannot be compared. This highlights one of the problems of studying the development trajectories of numerical competency in humans, namely the difficulty of devising experimental paradigms applicable at the same time to newborns, toddlers and infants. A second important limit of the research in humans and non-human primates is that for practical and ethical reasons it is very difficult to manipulate experience during development, which therefore precludes the possibility of disentangling the relative contribution of maturation and experience. The recent discovery that even simple organisms like fish and social insects are capable of numerical abilities similar to primates may pave the way to the use of new animal models in developmental research.

Single fish placed in an unknown environment show a strong tendency to join social companions and, if choosing between two shoals, they exhibit a preference for the larger one, an adaptive strategy that allows them to minimize the risks of predation [37], [38]. This spontaneous tendency has been recently used to explore the limits of numerical abilities in these phylogenetically distant organisms [30], [39]. Female mosquitofish discriminate groups differing by one unit up to 3 vs. 4 elements. They can also discriminate larger groups, at least up to 16 elements, provided there is a twofold or larger ratio between them. These capacities are shown even after the fish are prevented from using non numerical perceptual variables that co-vary with numerosity, suggesting that they can base quantity discrimination on pure numerical information [39]. Circumstantial evidence suggests that, like primates, fish may possess two separate mechanisms for representing small and large quantities. Fish could easily discriminate groups with ratios of 2∶3 or 3∶4 in the small quantity range, but not when larger numerosities were involved; the performance appeared ratio-dependent for large, but not for small numbers and a different combination of continuous variables affected discrimination in the two ranges [30].

In the present study we investigated the development of numerical discrimination in guppies (*Poecilia reticulata*). Due to a relatively short life-span and to independence at birth, guppies represent an excellent experimental model for studying the developmental trajectories of the capacity to discriminate small and large quantities. Four different experiments were performed. Experiment 1 was designed to assess the ability to discriminate small and large quantities at birth. Experiment 2 aimed to determine the upper limits of numerical discrimination in the small number range. Experiment 3 studied the influence of maturation and experience on large number discrimination. Experiment 4 was designed to determine if newborn and juvenile guppies retain the ability to discriminate quantities after being prevented from using non-numerical attributes of the stimulus.

## Results

### Experiment 1. Can newborn fish discriminate between social groups differing in numerosity?

In the first experiment we asked whether newborn guppies without any previous social experience showed the same ability as adults to discriminate between groups of peer fish differing in numerosity. We tested fish in discrimination between two small (2 vs. 3 fish) and between two large shoals (4 vs. 8 fish). Both discriminations are easily performed by adult fish [30]. Since toddlers are sensitive to numerical differences only at large ratios and their precision increases over development [29], we additionally tested newborn fish in a discrimination with a threefold ratio (4 vs. 12). Finally guppies were tested in comparisons between one number in the small quantity range and one large number (2 vs. 5 and 3 vs. 8).

**Figure 1:**
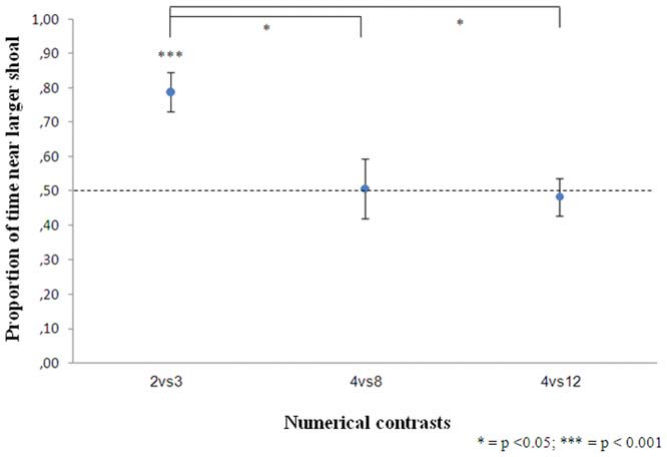
Results of experiment 1. Newborns spent more time near the larger shoal in the small quantity comparison while no choice has been reported in large quantity comparisons.

The position of the stimuli (right/left) did not affect fish preference in 2 vs. 3 (independent t-test, t(18) = 0.034, p = 0.973), 4 vs. 8 (t(18) = 0.071, p = 0.944) or 4 vs. 12 (t(18) = −1.423, p = 0.172). A significant choice of the larger shoal was found in 2 vs. 3 (one sample t-test, t(19) = 4.503, p<0.001); on the contrary, no preference was observed either in 4 vs. 8 (t(19) = 0.012, p = 0.990) or in 4 vs. 12 (t(19) = 0.133, p = 0.895, Fig. 1). An overall one-way ANOVA on the proportion of time spent near the larger shoal showed a significant difference between the three numerical contrasts (F(2,59) = 5.917, p = 0.005). Bonferroni post hoc tests revealed a difference between ‘2 vs. 3’ and the other numerical contrasts (‘4 vs. 8’ p = 0.014, ‘4 vs. 12’ p = 0.012), while no difference was found between the latter two (p = 1). In the additional test contrasting one number in the small quantity range and one large number, newborn guppies significantly selected the larger shoal (2 vs. 5 fish: mean ± std. dev.  = 0.642±0.056, t(19) = 2.616, p = 0.017; 3 vs. 8 fish: 0.670±0.210, t(19) = 3.425, p = 0.003).

At birth, guppies showed the ability to choose the larger shoal when the choice was between numbers in the small quantity range, 2 vs. 3 fish, but not when they had to choose between two large sets, 4 vs. 8 or 4 vs. 12 fish, even if the numerical ratio was larger in the latter cases. This suggests that in fish the capacity to discriminate among small quantities is innate while the capacity to discriminate large quantities should emerge later in development. However they were able to choose the larger shoal when they had to discriminate one number in the small quantity range from a number outside it (2 vs. 5 and 3 vs. 8 fish).

### Experiment 2. Limits of newborns' ability to discriminate between small quantities

Previous experiment provided information about a single numerical contrast in the small number range, 2 vs. 3 fish. In the second experiment we aimed to investigate the exact limit of the newborns' ability to discriminate between small shoals differing by one unit. The following numerical contrasts were presented: 1 vs. 2, 2 vs. 3, 3 vs. 4, 4 vs. 5 and 5 vs. 6.

**Figure 2:**
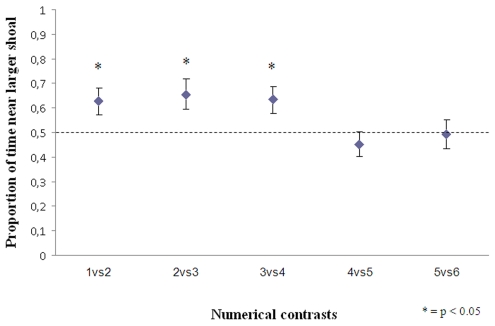
Results of experiment 2. Newborns proved to be able to discriminate shoals differing by one individual up to 4 units.

Fish spent more time near the larger shoal in 1 vs. 2 (t(19) = 2.424, p = 0.026), 2 vs. 3 (t(19) = 3.074, p = 0.006) and 3 vs. 4 (t(19) = 2.356, p = 0.029). No significant preference was observed in 4 vs. 5 (t(19) = −0.984, p = 0.338) and 5 vs. 6 (t(19) = −0.155, p = 0.878, Fig. 2). The difference between the three contrasts within the small quantity range (1 vs. 2, 2 vs. 3, 3 vs. 4) and the two contrasts involving larger numerosities (4 vs. 5, 5 vs. 6) is significant (ANOVA F(4,99) = 2.953, p = 0.024, planned contrasts t(95) = 28.804, p<0.001).

Thus at birth the capacity of guppies to discriminate between sets differing by one unit includes all numerical contrasts in the range 1–4 (1 vs. 2, 2 vs. 3, 3 vs. 4) but not contrasts involving larger numbers such as 4 vs. 5 and 5 vs. 6. In the small quantity range, the numerical abilities of newborn guppies appear the same as that shown by adult fish and primates [19], [30].

### Experiment 3. Development of the large quantity discrimination

Experiments 1 and 2 show that one-day-old guppies can distinguish the larger of two small quantities, whereas they do not select the larger shoal when two large quantities are presented even when the ratio is large. Because adult fish can easily do this discrimination [12], [30], [37], in the present experiment we investigated the development of large quantity discrimination by testing fish at three different ages (1-, 20- or 40-day-old). In addition, to assess the role of experience, half of the guppies were reared in large groups with the possibility of seeing shoals of variable numerosities and half were reared in pairs, without the possibility of seeing more than one fish at a time.

**Figure 3:**
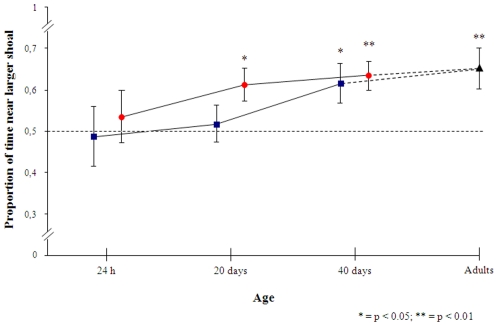
Results of experiment 3. Fish ability to discriminate between 4 and 8 increases in precision over development. *Circles* with numerical experience in large shoals, *squares* without numerical experience reared in pairs. The score of 14 adult female guppies (*triangle*) is also shown for reference (source: manuscript submitted).

Among guppies reared in pairs (without numerical experience), both 1-day- and 20-day-old fish show no significant preference (respectively t(19) = 0.103, p = 0.919 and t(23) = 0.552, p = 0.586), whereas we found a significant preference for the larger group when testing 40-day-old fish (t(21) = 2.413, p = 0.025). Fish reared in groups (with numerical experience) did not select the larger group when 1-day-old (t(31) = 0.539, p = 0.593), but did so at 20 and 40 days of age (respectively t(23) = 2.735, p = 0.012 and t(21) = 3.861, p = 0.001, Fig. 3).

Data were analyzed by 2 (Numerosity: smaller/larger shoal)×2 (with/without numerical experience)×3 (Age: 24 h/20 d/40 d) ANOVA. A significant effect was observed for the factors Numerosity (F(1,128) = 10.788, p<0.001), Age (F(2, 128) = 4.746, p = 0.010) and Experience (F(1, 128) = 6.144, p = 0.014). No interaction was significant (all ps >0.05).

Thus at forty days, before the onset of sexual maturation, young guppies were able to discriminate 4 vs. 8 fish like adult fishes and there was no difference between subjects with different social experience. However fish raised in pairs (with no experience of social groups) and fish raised in large social group (with the possibility of observing groups of different numerosity) differed in the onset of large number discrimination that appeared earlier in the latter treatment. Combined with the results of experiment 1, these results suggest that the ability to discriminate number 1–4 is displayed immediately at birth while the capacity of discriminating larger numbers emerges later in consequence of both maturation and social experience.

### Experiment 4. Can young guppies discriminate quantities by using numerical information only?

In previous experiments the ability of newborn and young guppies to discriminate among numerosities closely resembles that observed in adult primates in comparable tasks. Yet, numerosity normally co-varies with several other physical attributes, and organisms can use the relative magnitude of continuous variables such as the total area of the stimuli or the density of elements to estimate which group is larger/smaller. In this experiment we used a modification of the ‘item by item presentation’ procedure previously adopted in infant and monkey studies [19], [40], [41] to investigate whether fish can discriminate between quantities by using numerical information only. Subjects could choose between one large and one small group of companions but they could only see one fish at a time, thus preventing the possibility that they could use perceptual features of the shoal to select the larger set.

Two different numerical contrasts were presented: a small quantity task (2 vs. 3) to newborns and a large quantity task (4 vs. 8) to juveniles. Both tests were controlled for non numerical cues provided by the shoal, namely the overall space occupied by the shoals, the density of the fish and the total surface from which stimulus fishes were visible to the subject (visibility space).

#### 4a. Small quantity discrimination

The data were analyzed by 2 (Numerosity: smaller/larger shoal)×2 (Position of the large shoal: right/left)×3 (Continuous variable control: overall space/density/visibility space) ANOVA. Subjects spent significantly more time near the larger shoal (time spent near the larger shoal, mean ± std. dev.: 1804±890 seconds, time spent near the smaller shoal: 1060±760; F(1,42) = 10.999, p = 0.002). The subjects' preference was not influenced by the position of the larger shoal (F(1,42) = 1.252, p = 0.270) or by which continuous variable was controlled for (F(2,42) = 2.742, p = 0.076). No interaction was significant (all ps >0.05).

#### 4b. Large quantity discrimination

The data were analyzed in a 2 (Numerosity: smaller/larger shoal)×2 (Position of the larger shoal: right/left)×3 (Continuous variable control: overall space/density/visibility space)×2 (Experience: reared in pairs/reared in groups) ANOVA. Subjects spent significantly more time near the larger shoal (time spent near the larger shoal: 1992±868, time spent near the smaller shoal: 1296±840 seconds, F(1,60) = 12.648, p = 0.001); fish reared in groups spent more time near the stimuli than subjects reared in pairs (F(1,60) = 8.527, p = 0.005). Subjects' preference was not influenced either by the position of the larger shoal or by which continuous variable was controlled for (respectively F(1,60) = 0.030, p = 0.864; F(2,60) = 2.930, p = 0.061). No interaction was significant (all ps >0.05).

## Discussion

Previous studies have shown that when adult fish are given the choice between two social groups they choose the larger and discriminate groups differing by one unit up to 3 vs. 4 elements. They can also discriminate larger groups, provided there is a twofold or a larger ratio between them (e.g. 4 vs. 8 or 8 vs. 16 but not 8 vs. 12 fish [12], [30]). In Experiment 1 newborn guppies tested in similar conditions chose the larger shoal when the choice was between numbers in the small quantity range, 2 vs. 3 fish, but not when they had to choose between two large sets, 4 vs. 8 fish, although the ratio was larger in the latter case. In the second experiment we showed that, at birth, the capacity to discriminate sets differing by one unit is the same as that shown by adult fish and non-human primates and includes all numerical contrasts in the range 1–4 (1 vs. 2, 2 vs. 3, 3 vs. 4) but not contrasts involving larger numbers such as 4 vs. 5 and 5 vs. 6. Thus, in fish the ability to discriminate sets in the small number range appears to be innate and displayed immediately at birth, while the discrimination of large quantities develops later. Experiments on infants showed that they require larger numerical ratios than adults to discriminate large quantities (a threefold ratio at birth, a twofold ratio in 6-month-olds, a 2∶3 ratio in 10-month-olds and a 8∶9 ratio in adults). It is therefore possible that newborn guppies can discriminate large numerosities but that they require larger differences compared with adult fish. When tested with a threefold ratio (4 vs. 12 fish), newborn fish still failed the discrimination. It is worth noting that when tested with numbers in the small quantity range, newborns not only discriminate a twofold ratio (1 vs. 2) but also much closer ratios such as 2∶3 and 3∶4 (2 vs. 3 and 3 vs. 4).

Given that adult guppies easily discriminate 4 from 8 fish (unpublished data), in the third experiment we asked when this capacity develops, by comparing fish of three different ages. Guppies start to socially interact with peers immediately after birth and the experience of choosing between alternative groups of different numerosity is probably frequent during development. Hence we also investigated the role of previous experience with numbers by raising fish in two treatments. In one, fish had a normal experience with numerous peers and hence the chance to familiarize themselves with sets of different numerosity. In the other, they were raised with a single peer so that they could socially interact but never experience groups of fish. The results clearly show that 40-day-old guppies both with and without numerical experience discriminate 4 from 8 fish, while among 20-day-old guppies this capacity was only observed in fish with experience of numbers. Discrimination of large numerosities thus emerges later in development and appears to be modulated by specific experience with social groups. The twofold ratio discriminated by young fish is also the threshold of large number discrimination in mature fish [12], [30] including guppies (unpublished data). Therefore by the third week, well before the onset of puberty (normally around 12–15 weeks in lab conditions), juvenile guppies develop the full range of numerical capacities observed in an adult. Guppies are livebearing and their newborns are quite advanced in development compared with other vertebrates such as primates or birds. Yet while nestlings and primate babies rely entirely on their parents for feeding and protection, young guppies get by on their own and their survival depends on skills such as rapidly determining which group offers the best protection from predators.

One may argue that the lack of choice by newborn fish in tests involving large shoals in Experiments 1 and 3 might be due to age-related differences in social motivation rather than to cognitive differences between newborns and older fish. It is possible, for example, to hypothesize that newborn guppies are able to discriminate 4 from 8 fish but, at this early age, they tend to avoid very large groups, a tendency that would disappear as they grow older. The choice of the larger group in the comparison of 3 vs. 8 fish in Experiment 1, however, seems to exclude this interpretation. The fact that guppies discriminate 3 vs. 4 and 3 vs. 8 fish, but not 4 vs. 8 fish seems to indicate that the small number range of guppies at birth may be limited to numbers 1–3 as in 6-month-old infants and birds [42], [43]. In this view they would be able to estimate when a number exceeds the small number range, although they would be incapable to discriminate among numbers exceeding that range, whatever their difference. So, newborn fish would recognize that 4 is larger than 3 as well as that 8 is larger than 3, but they would be unable to discriminate which one between 4 and 8 fish is the larger set.

Numerosity normally co-varies with several other physical attributes, and an animal can use the relative magnitude of continuous variables such as the total area occupied by objects or the density of elements to provide quantity judgments without necessarily being capable of numerical representation [44], [45]. For example, in the situation of our experiments, a larger shoal normally occupies a larger space than a smaller one and if forced to occupy the same space, it shows higher fish density. Therefore, when investigating numerical competence in an organism, it is important to investigate if the discrimination between two sets of objects is maintained after the use of non-numerical cues has been prevented. This was shown to occur for two different tasks in adult fish [11], [39], but there is currently no evidence that young fish can also rely on the sole numerical information. One strategy that was often employed to exclude the use of continuous extent in studies with infants and monkeys is the sequential presentation of items within each set, so that subjects cannot have a global view of the entire contents of the sets [19], [40], [41]. In Experiment 4 we adapted the ‘item by item’ procedure to investigate whether fish can discriminate between small quantities by using numerical information only. Subjects could choose between two different groups of fish but the apparatus was modified in a way that they could only see one fish at a time, thus preventing the possibility that they could use perceptual features of the shoal to select the larger set. A significant preference for the larger shoal was found in discrimination both between small quantities (2 vs. 3) and between large quantities (4 vs. 8) and we found no statistical difference when we matched the two groups for the overall space occupied by the stimulus fishes, for their density or for the total surface from which stimulus fishes were visible to the subject.

One may argue that in experiment 4 guppies may have stored the perceptual features, such as the area, of each individual stimulus fish in short-term memory, and then used this information rather than the actual number of fish in each shoal to choose the larger one. In a study using the ‘item by item’ method in 12-month-olds [16], after seeing crackers placed sequentially into two containers, infants were allowed to crawl and choose one of the containers. Infant chose the larger quantity with comparisons of 1 versus 2 and 2 versus 3 although they failed with larger numbers. However when crackers were of different sizes, the choice was determined by total surface area or total volume. The authors argue that their results are better explained by assuming that infants rely on object-file representations, comparing mental models via total volume or surface area rather than via one-to-one correspondence between object files. However in their experiment the reward was food and it is not unexpected that natural selection had shaped the quantificational systems in order to maximize the amount of food (i.e. calories) retrieved rather than the number of pieces and therefore we expect the choice of the larger volume of crackers irrespective of the fact that 12-month-olds can or cannot discriminate quantities using the sole numerical information. In experiment 4 one must assume that guppies stored the perceptual features of all fish from one side, then mentally summed these areas and compared this information with the sum of fish areas of the opposite side. Though possible, in our view this would represent a less parsimonious explanation requiring that fish form some representation of each stimulus fish including its perceptual features as well as that they possess the capacity to memorize and sum up to eight different areas.

The developmental dissociation observed in this study, with large number discrimination appearing later and being influenced by experience, suggests the existence of two separate underlying mechanisms. Traditionally, it is assumed that adults possess distinct non-verbal systems of numerical representation, an object file system used for small quantities and an analog magnitude system that allows representing larger quantities [46], [47]. Based on comparative and developmental evidence, some authors have suggested that distinct mechanisms for large and small number representation may operate in infants, non-human primates and possibly in other animals too [17], [19], [48]. However, recent publications have sparked debate over whether adults, infants and non-human primates represent small numbers via an object file system, by providing experimental evidence that an analog magnitude system is used for representing both small and large numbers. In one study, in which 3-year-old children were asked to match a sample stimulus to one of two choice stimuli, a significant effect of numerical ratio was found in discrimination of quantities 1–4, similar to the ratio effect observed with large sets [49]. Evidence for analog numerical representation in the small quantity range was provided for adults too. College students were presented with an Arabic numeral and asked to press a key for the specified number of times while verbal counting was suppressed. No significant difference was found between the regression slopes of the data for numbers in the small quantity range (2–5) and the slopes for numbers beyond that range [50].

Some evidence on chimpanzees, macaques and lemurs also suggest that they use a single system over the whole numerical range [51], [52], [53]. Cantlon and Brannon [53], testing number-experienced and number-naïve rhesus monkeys in a delayed match-to-sample task similar to that used with children [49], found a significant effect of numerical ratio on accuracy for numerosities 1–4, an indication that non-human primates may rely on analog magnitude representations for both small and large numbers. In this study, experienced and naïve monkeys showed differences in their ratio-dependency, raising the possibility that analog representation of small sets may be modulated by individual and contextual variables.

While these studies convincingly demonstrate that in some experimental conditions subjects rely on the analog magnitude system to represent quantities in the small number range, they do not exclude the possibility that different systems are used for representing small numbers in other circumstances. The two supposedly underlying mechanisms, the object file system and the accumulator system, greatly differ in speed, accuracy and cognitive load [54], [55] and one can argue that small sets may be represented by different mechanisms, depending on factors such as the type of task, the nature of stimuli and previous experience. Indeed, numerous observations indicate humans and non-human primates often represent small and large numerical quantities in qualitatively different ways. Various studies have reported that adults performance is extremely fast and accurate in the range 1–4, but outside this range, as numerosity increases, each additional item has a substantially greater cost in terms of reaction time and accuracy [21], [56], [57], [58]. In contrast with the findings of Cordes and collaborators [50], a new experiment with adults has shown that accuracy was ratio-dependent for large numbers, while there was a clear violation of Weber's law in the range 1–4 [46] and a study of event-related potentials has provided the first neurophysiological evidence of separate mechanisms for processing large and small numbers [59]. Recently a patient was described in which counting was impaired while accuracy in the range 1–4 was preserved [60] and a study of infants with Williams syndrome reports a specific impairment in large number discrimination while the discrimination capacity in the small number range was unaffected [61]. A previous study reports that chimpanzees are quite accurate and fast responding to numerosities 1–3, while for numerosities larger than 4 they show a monotonic increase in reaction time [24] and evidence for a representation of small sets on the basis of one-to-one correspondence has been provided for other non-human primates [19], [62].

In sum, several lines of evidence converge in indicating that both human and non-human primates possess separate systems of representation for small and large numbers, although convincing literature indicates that the analog magnitude system may sometimes be recruited to represent numbers in the small quantity range too. Our findings reinforce the view of distinct numerical systems and additionally suggest that separate processing of small and large numbers may have a long evolutionary history.

Recent findings in the literature have reported that infants apparently fail to compare small (<4) and large values [18], [63]. In fact, while they consistently succeed at discriminating values within the large number range (e.g., 8 vs. 16), and values within the small number range (e.g., 1 vs. 2), they are unable to discriminate across small and large values (e.g., 2 vs. 4). The explanation usually given for this phenomenon is that because infants represent large numbers using analog magnitudes and small numbers via object files this generates an incompatibility in representational formats that prevents comparison and hence determines the failure in the discrimination [16], [18]. Similarly the failure to discriminate large from small values was recently reported for adult guppies (unpublished data). This raises the question of why newborn guppies unlike adults are able to discriminate across numerical domains as observed in 2 vs. 5 and in 3 vs. 8 sets. A possible explanation is that at birth guppies lack the analog magnitude system, thus by-passing potential conflict between representational formats generated by activation of large- and small-number systems. This conflict would eventually arise in adults, after the maturation of the analog magnitude system.

The observation that adult fish and infants are unable to compare values across two numerical ranges could potentially provide an alternative interpretation of our results. One may argue that if we posit that, unlike infants and several non-human species [10], [43], [64], for guppies the number 4 belongs to the small number range, the failure to discriminate 4 from 8 at birth might reflect the inability to compare small and large values rather than the lack of the analog magnitude system. The capacity to discriminate small from large sets would eventually emerge in juveniles allowing them to discriminate 4 from 8 items. This interpretation is inconsistent with the evidence reported above that, quite the opposite, newborn fish can compare values across different numerical ranges while this ability is apparently lacking in adult guppies. Recently Cordes and Brannon [65] reported that 7-month-olds can successfully discriminate small from large sets when the difference is very large as in 2 vs. 8 items. This finding also argues against the alternative interpretation of our results since in our study newborn were unable to perform the 4 vs. 12 discrimination that has a very large numerical ratio (1∶3) while they succeed in two cross-range discriminations, 2 vs. 5 and in 3 vs. 8 with a smaller numerical ratio (1∶2.5 and 1∶2.67 respectively).

Our experiments do not provide an explanation of why in fish the capacity to discriminate small numbers is in place at birth, while the capacity to discriminate large numbers emerges later as a result of both maturation and social experience. Several authors believe that the capacity of rapid and accurate numerical judgments on small sets of items is based on an object-tracking system [21], [22]. The object-tracking mechanism did not originate as a system of numerical representation. Rather, it is believed to be an evolutionarily ancient system allowing individuals to track up to 3–4 objects in parallel even if these are moving in space, provided they remain in view or undergo brief periods of occlusion [21], [66]. Such a system could secondarily be co-opted for numerical tasks when numerosities involved are small. To survive in their environment, guppies must be able to track multiple objects such as live prey, potential predators or social companions and these abilities must be in place from birth. It thus makes sense that guppies are born equipped with mechanisms similar to the object-tracking system hypothesized for humans. Even if true numerical mechanisms emerge only later in development, newborn guppies could use their object-tracking system to solve simple numerical tasks like deciding which social group is larger.

An almost complete lack of developmental data in literature precludes comparison of guppies with other species. The only possible comparison is with our own species, that is however phylogenetically and ecologically very distant from fish. Very little is known about small number representation before six months of age when infants normally discriminate two from three items [42], [63]. One exception is the early study by Antell and Keating (1983) in which, using a habituation/dishabituation paradigm, they observed that newborns (age range, 21–44 hours) were able to discriminate 2 from 3 dots but not 4 from 6 dots [67]. In another study that investigated whether 4-day-old infants discriminated syllables with different numbers of consonants-vowels, infants discriminated 2 vs. 3 items but not 4 vs. 6 items [68]. These evidences are compatible with the possibility that, as in guppies, human ability to discriminate number within the small number range might be place at birth.

As regards the large number discrimination, it is clearly present at six months and steadily increases in precision till the adulthood [1], [32], [34]. Recently Izard and collaborators (2009) documented that two-day-olds look longer at a visual arrays of objects when their number matched the number of syllables they have heard before [35]; this suggests that the approximate numerical system may be present very early in our species although at birth it appears more imprecise than in six- or nine-month-olds, requiring at least a threefold numerical ratio.

In conclusion, despite some differences, this study highlights several similarities between the numerical systems of fish and primates. In particular some of our results reinforce the view that there might be two distinct numerical systems in fish too. More research is necessary, both in primates and in fish, before the similarities suggested by this study are confirmed. In particular, for fish, research on different sensory modalities and with different paradigms is desirable. Recently it has been reported that fish can be trained to discriminate between sets containing different numbers of geometric figures in just a few days, a paradigm that has a great potential for future investigation of the development of numerical cognition in these organisms [11].

At this stage of the research, we should prudently consider the possibility that similarities between fish and primates in numerical capacities may be merely due to a coincidence or to similar evolutionary constraints acting on different organisms. Nonetheless, the numerous parallels between primates and fish shown in the present and in other studies raise the intriguing possibility that sophisticated numerical concepts of adult humans may be rooted in numerical systems that appeared more than 450 million years ago, a question that merits further investigation.

## Materials and Methods

### Experiment 1. Can newborn fish discriminate between social groups differing in numerosity?

#### Subjects

Sixty newborn guppies were used as subjects for the experiments. Twenty fish were tested in a 2 vs. 3 comparison, 20 in 4 vs. 8 and 20 in 4 vs. 12. In addition we tested guppies in comparisons between one number in the small quantity range and one large number, twenty in the 2 vs. 5 and twenty in 3 vs. 8 comparison. Guppies are viviparous and give birth to fully developed offspring that are completely independent and display a full social repertoire [69]. To prevent any social experience by newborn fish, females close to parturition were singly placed in nursery tanks and the light was switched off as parturition initiated. Fry used as subjects were collected and individually placed in 1 litre tanks for 24 hours to allow a complete recovery from birth. Fry used for stimulus shoals were of similar age but kept in a group. Subjects were tested only once.

#### Apparatus

**Figure 4:**
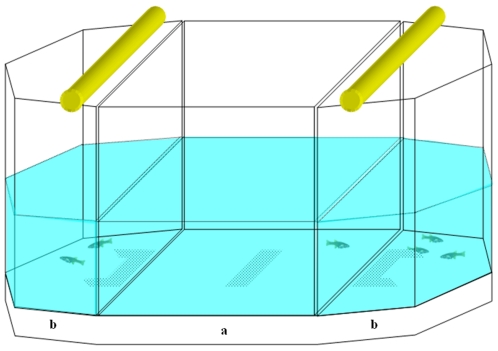
Schematic representation of the experimental apparatus used in experiment 1. a) subject compartment, b) stimulus compartments.

The apparatus was a small scale version of those used to study numerical discrimination in adult fish [30], [38] and consisted of a small tank subdivided into three adjacent compartments (Fig. 4). A central rectangular ‘subject compartment’ (20×19×25 cm) housed the test fish. At the two ends, two smaller ‘stimulus compartments’ were shaped as semi octagons (6.3 cm each side) and faced the subject compartment. Each stimulus compartment was lit by one fluorescent lamp with water maintained at a temperature of 25±2°C. The tank was externally covered with white plastic to prevent stimulus fish and subjects from seeing outside. A video camera was suspended about 1 m above the test tank and used to record the position of the subject during the tests.

#### Procedure

Stimulus fish were introduced in the lateral compartments 10 min prior to the test. The subject was introduced into the middle of the ‘subject compartment’ and allowed to choose for 15 min. For each numerical contrast half of the tests had the larger group on the left and half on the right. Subjects that did not visit each stimulus sector at least three times or spent less than the 50% of the time in the choice areas were considered inactive; they were discarded and replaced by other fish.

From video recordings we calculated the time spent by the subject shoaling within a distance of 4 cm from the glass facing the stimulus compartments (choice area). The observer of this video was blind with respect to the aim of the experiment. The dependent variable was the proportion of time (sec) spent close to the larger shoal. Significant departures from chance level (50%) were estimated by one-sample two-tailed t-tests. Frequencies were arcsine (square root)-transformed [70]. Mean ± SD are provided. Statistical tests were carried out using SPSS 17.0.

### Experiment 2. Limits of newborns' ability to discriminate between small quantities

One-hundred 24-hour-old fish were used as subjects, 20 for each numerical contrast (1 vs. 2, 2 vs. 3, 3 vs. 4, 4 vs. 5, 5 vs. 6). To avoid possible stress from social isolation and allow normal social development in this and the subsequent experiments, fish were kept in pairs in 4 litre tanks from birth to the time of the test. This allowed the subjects to socially interact but prevented them from seeing groups of fish before the test. The apparatus and the procedure were identical to those of Experiment 1.

### Experiment 3. Development of the large quantity discrimination

A total of 144 individuals were used as subjects. They were assigned to six different conditions. Subjects could be tested at three different ages (1-, 20- or 40-day-old) and two rearing conditions, with no experience of groups (in pairs) or with normal social experience (13–15 similar aged fish with 2 adults in a 60 l tank).

The same procedure and apparatus as in Experiment 1 was used. The only exception was the enlarged size of the stimulus compartments when 40-day-old fish were tested (7.3 cm each side instead of 6.3 cm). All the subjects were tested in the same large quantity comparison, 4 vs. 8.

### Experiment 4. Can young guppies discriminate quantities by using numerical information only?

#### 4.a Small quantity discrimination

**Figure 5:**
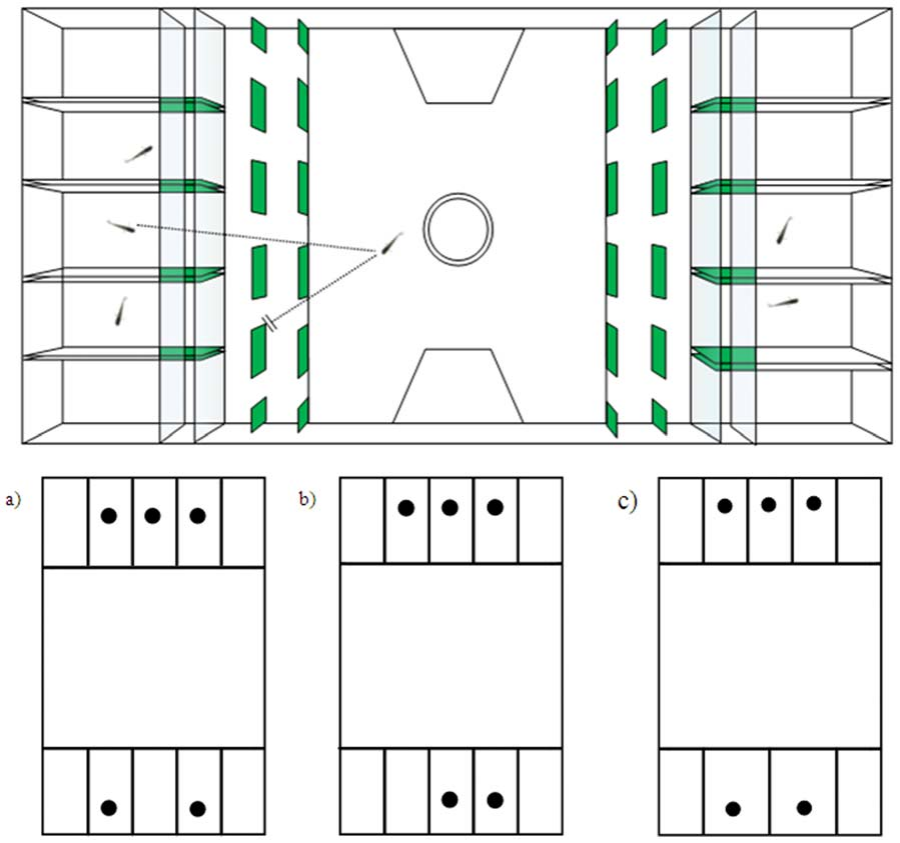
Experimental apparatus used in experiment 4a. Subjects could only see one stimulus fish at a time from any position of its compartment. To reduce the access to non-numerical cues, one third of the sample were tested matching the overall space occupied (a), one third matching the density (b) and one third matching the surface from which a fish was visible (c). The apparatus for Exp. 4b was similar but enlarged in size in order to adapt to the larger size of juvenile fish.

A total of 48 one-day-old fish were used as subjects. Fish were reared in pairs before the test and used only once. The experimental apparatus was similar to that used in a previous study [39] with a closely related species, the eastern mosquitofish, and was composed of a tank subdivided into three adjacent sectors (Fig. 5). The central one, the ‘subject sector’, was an hourglass-shaped sector of 18.5×15.5 cm consisting of a corridor interconnecting two identical choice areas (5×15.5 cm). At the two ends there were two sectors, ‘stimulus sectors’, facing the subject sector. Each stimulus sector (7.5×15.5 cm) was subdivided into 5 identical compartments (6×2.6 cm) by translucent walls that prevented stimuli from seeing each other. Only the three central compartments were used. To avoid the subject seeing more than one stimulus at a time, in each choice area 12 vertical green screens (1.6×6 cm) were placed, set in a grid of 6×2. In this way the subject could only see one stimulus at a time from any position in its sector.

The subjects could choose between 2 and 3 fish. To control for continuous variables, fish were tested in three different conditions (16 subjects per condition), which differed in the spatial position of the stimuli. In the first condition, we matched the overall space occupied by the stimuli by equalling the distance between the two most lateral fish on the two sides (Fig. 5a); in the second condition we matched the density by keeping a constant distance between each stimulus fish (5b). In the third condition we matched the surface from which a fish was visible to the subject (5c). To obtain this, on the side containing two fish, the compartments containing stimuli were enlarged to 3/2 of normal width (4 cm). We accordingly modified the number and the size (2.4×6 cm) of the opaque screens in front of the smaller shoal, positioning them in a grid of 5×2.

The subject was introduced into the middle of the subject sector and it was allowed to explore the apparatus for 120 min. After this period the subject's position was recorded for 60 min. Shoal preference was calculated as the time spent in the choice area. Movements during the first two hours were recorded and those fish that did not visit each stimulus tank at least 14 times in that period were discarded and replaced.

#### 4.b Large quantity discrimination

A total of 72 juvenile fish were used as subjects. Fish belong to two different groups reared in the same conditions as Experiment 3. Thirty-six were 20-day-old fish reared in groups of 13–16 individuals per tank and 36 were 40-day-old fish reared in pairs. Each fish was used only once.

The experimental apparatus was similar to that used in Experiment 4a. The ‘subject sector’ was enlarged (48×35.6 cm) in order to adapt to the larger size of these subjects. Moreover, each ‘stimulus sector’ (48×13.5 cm) was subdivided into 8 identical compartments (4×12 cm); to avoid the subject seeing more than one stimulus at a time in each choice area, 16 vertical green screens (2.7×8 cm) were placed, set in a grid of 8×2. Subjects were given a choice between 4 and 8 conspecifics. As in Experiment 4a, one third of the subjects (twelve 20- and twelve 40-day-old fish) were tested matching the overall space occupied, one third matching the density and one third matching the surface from which a fish was visible to the subject. The procedure was identical to Experiment 4a.
